# Stereotactic Body Radiation Therapy for Hepatocellular Carcinoma in Child-Pugh Class C As Bridge Therapy Before Liver Transplantation

**DOI:** 10.7759/cureus.71654

**Published:** 2024-10-16

**Authors:** Atsuto Katano, Yuki Nozawa, Masanari Minamitani, Hideomi Yamashita

**Affiliations:** 1 Radiology, University of Tokyo Hospital, Tokyo, JPN

**Keywords:** child-pugh class c, hepatocellular carcinoma, liver neoplasms, liver transplantation, stereotactic body radiation therapy

## Abstract

Hepatocellular carcinoma (HCC) is a rising global health concern, frequently developing in patients with cirrhosis. Stereotactic body radiotherapy (SBRT) offers an effective treatment option for localized HCC, utilizing advanced imaging and planning technologies to accurately target tumors while sparing healthy tissues. It serves as an alternative when other locoregional therapies are contraindicated. We present the case of a 58-year-old male with HCC exceeding 5 cm and classified as Child-Pugh Class C. Following a failed attempt at transarterial chemoembolization (TACE) due to deteriorating liver function, SBRT was selected as an alternative bridging therapy. The patient received a total dose of 30 Gy in five fractions. Throughout the treatment, he experienced no significant toxicity, and post-SBRT imaging indicated gradual tumor regression. Seven months post-SBRT, the patient successfully underwent deceased-donor liver transplantation. Pathological examination of the explanted liver revealed necrotic nodules, indicating a positive response to SBRT.

## Introduction

Hepatocellular carcinoma (HCC) mortality is rising globally, with a significant shift from virus-related to non-viral causes, such as alcohol and metabolic liver diseases [1,2]. HCC often arises in patients with cirrhosis, where it is the leading cause of death among cirrhosis patients [3]. Although liver transplantation is the most definitive treatment, eligibility is frequently constrained by tumor size, disease progression, and liver function [4]. The Milan criteria limit transplant candidacy to patients with a single tumor no more than 5 cm in diameter [5]. For those with tumors exceeding these criteria, bridging therapies are crucial to control tumor progression and maintain eligibility while awaiting transplantation.

Conventional radiation therapy has traditionally minimized the impact on surrounding healthy tissues by delivering relatively low doses of radiation over an extended period. In contrast, stereotactic body radiotherapy (SBRT) delivers precise and potent radiation to tumors while sparing surrounding normal tissues, thanks to advanced image-guided technologies, innovative treatment planning, and rigorous quality assurance protocols [6]. SBRT has been utilized as a treatment option for localized HCC patients when other locoregional therapies are contraindicated. According to a meta-analysis conducted by the International Stereotactic Radiosurgery Society, clinical outcomes and toxicity rates following SBRT for HCC were evaluated across 17 studies involving 1889 patients [7]. The analysis revealed 3- and 5-year overall survival rates of 57% and 40%, respectively, and local control rates of 84% and 82%.

This case report represents the effective use of SBRT as a bridging therapy for liver transplantation in a patient with HCC exceeding 5 cm and advanced cirrhosis classified as Child-Pugh Class C.

## Case presentation

**Figure 1:**
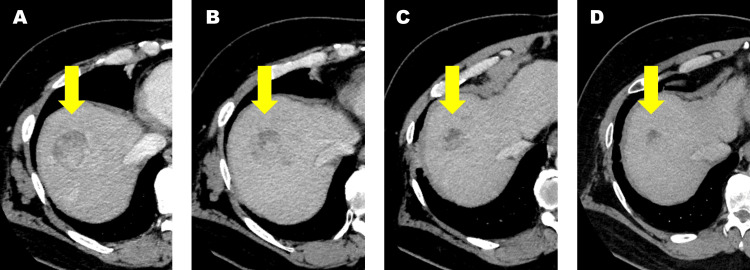
A CT scan of the abdomen. CT images of the lesion located in segment 8 before radiotherapy (A), and at one month (B), three months (C), and six months post-treatment (D). Yellow arrows indicate the targeted lesion.

A 58-year-old male was referred to our department for SBRT as a bridging therapy for liver transplantation. He was classified as Child-Pugh Class C and presented with HCC exceeding 5 cm in diameter at segment 8 (Figure 1A).

Due to the tumor size, he was ineligible for liver transplantation at that time. The patient showed signs of decompensated liver disease, with no ascites and mild hepatic encephalopathy. Laboratory tests revealed: Albumin: 2.8 g/dL, Total Bilirubin: 4.9 mg/dL, Prothrombin Time: 16.8 seconds (prothrombin time activity percentage: 46%), and Ammonia: 233 µg/dL. He had been registered on the liver transplant candidate list for two years and had a history of multiple transarterial treatments to control HCC rupture.

Initially, transarterial chemoembolization (TACE) was planned for this lesion at segment 8; however, deteriorating lab results indicated poor liver reserve function, classified as Child-Pugh C, presenting an extremely high risk that made TACE inappropriate. Additionally, reduced hemostatic ability contributed to the cancellation of the procedure. Given the advanced cirrhosis, surgical resection was also not an option. Despite these challenges, the patient was evaluated and remained a suitable candidate for liver transplantation. However, bridging therapy was necessary to prevent tumor progression while awaiting a donor liver.

**Figure 2:**
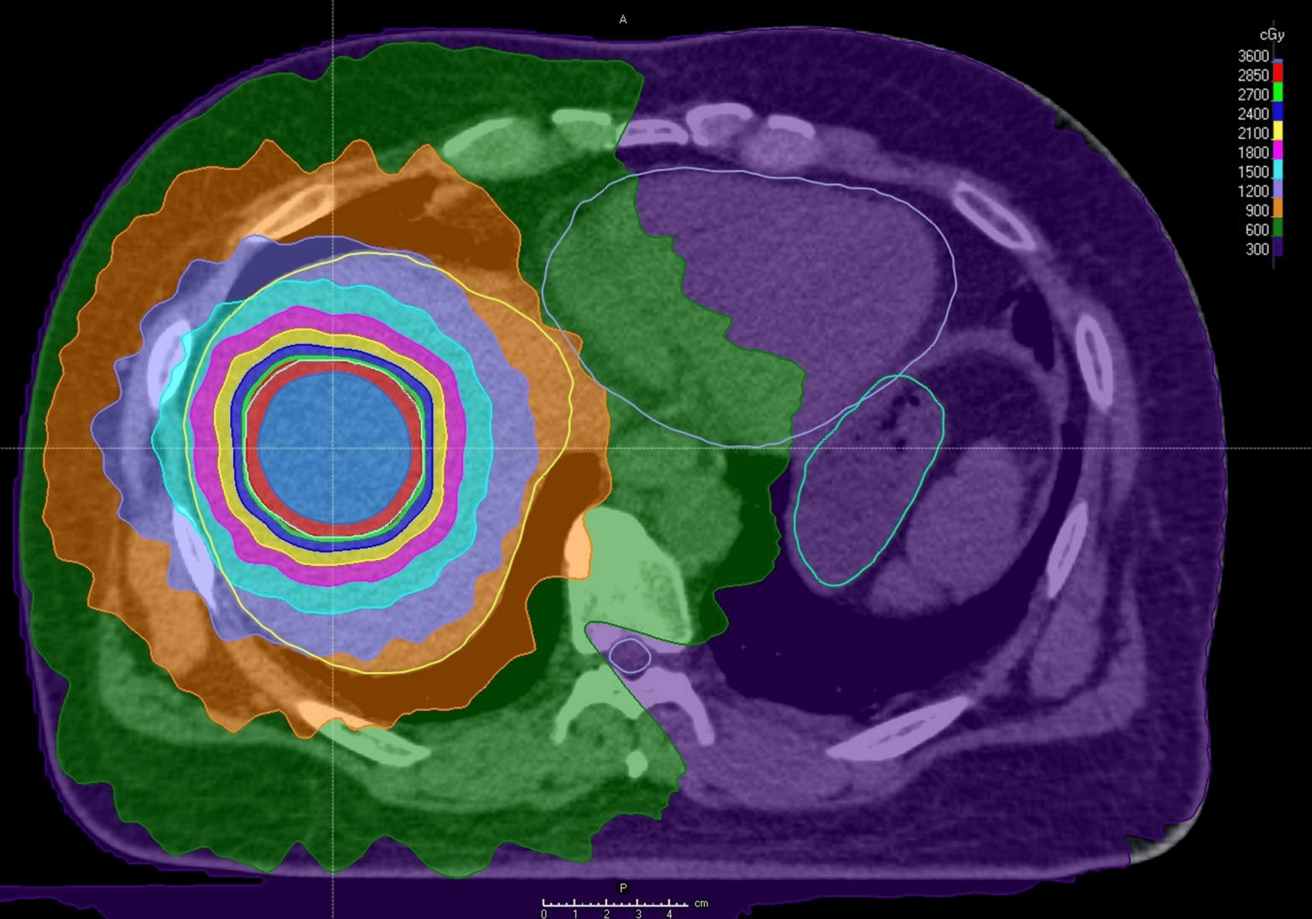
Treatment plan overlaid on a CT scan of the abdomen. Stereotactic body radiotherapy treatment plan with the prescribed radiation dose shown in the upper right region. The area colored in red represents the region where at least 95% of the prescribed dose is delivered, and the tumor is located within this area. Organs at Risk (OARs): the stomach is outlined in sky blue, the heart in lavender, and the liver in yellow. The outlines around the organs highlight these OARs.

After a multidisciplinary tumor board discussion, SBRT was selected as the most appropriate bridging therapy due to the patient’s poor liver reserve and the potential for SBRT to control tumor growth while minimizing further hepatic decompensation. The gross tumor volume (GTV) was delineated by referencing gadolinium-ethoxybenzyl-diethylenetriamine penta-acetic acid-enhanced MRI images to accurately contour the tumor volume. The clinical target volume (CTV) was equal to the GTV, and the planning target volume (PTV) added an isotropic 5 mm margin in all directions to the CTV. The patient underwent SBRT, receiving a total dose of 30 Gy in five fractions, administered twice per week over a duration of 13 days. The prescribed dose covered 95% of the PTV volume, corresponding to the 54% isodose line of the maximum dose (Figure 2).

Our institution utilized a multiple breath-hold technique to confirm diaphragm dome position without the use of implanted fiducial markers, as detailed in previous studies [8-11]. The parameters for the organs at risk in this radiation therapy are shown in Table 1.

**Table 1:** Dose summary table in stereotactic radiotherapy for the present study. Dose parameter in the present study: 'Dxx' refers to the dose received by a certain percentage of a specified volume of the target. PTV: Planning Target Volume.

Target	Volume (cm³)	D98 (cGy)	Mean (cGy)	D50 (cGy)	D2 (cGy)
Duodenum	69.67	0	16	3	71
Heart	1208.91	6	197	60	759
Kidney_L	314.07	0	3	3	6
Kidney_R	316.00	0	20	10	74
Normal Liver	1229.90	44	948	753	3438
PTV	100.96	2903	4231	4251	5599
Spinal Cord	- (Not Assessed)	0	89	11	536
Stomach	866.65	7	97	28	517

Throughout treatment, the patient experienced no adverse toxicity and tolerated the therapy well.

Following SBRT, regular imaging demonstrated gradual tumor regression over a 6-month period (Figures 1B-1D). There was no significant worsening of liver function post-SBRT until deceased-donor liver transplantation. Seven months after SBRT, the patient successfully underwent deceased-donor liver transplantation, and post-transplant pathology revealed necrotic nodules in the liver, indicating a favorable response to SBRT. At the six-month follow-up after the transplantation, the patient shows no evidence of HCC recurrence.

## Discussion

The case of bridging liver transplantation with SBRT for a patient with HCC larger than 5 cm and Child-Pugh Class C cirrhosis presents a unique therapeutic challenge. SBRT offers a viable alternative for local control of HCC in such high-risk patients while they await liver transplantation.

SBRT has proven to be an effective bridging therapy for patients awaiting liver transplantation with unresectable HCC. Lee VH et al. reported in a phase 2 trial that 32 patients with a Child-Pugh score up to B8 achieved an objective response rate of 87.5% for lesions after SBRT as bridging therapy for liver transplantation [12]. Among the 20 patients who subsequently received a deceased donor liver transplant, 15 exhibited a pathologic complete response in the liver explants.

In patients with advanced liver disease classified as Child-Pugh Class C, therapeutic options are limited due to severely compromised liver function [13]. Transarterial therapies or surgical resection are often infeasible, necessitating alternative, less invasive treatments. A major concern with radiation therapy in this population is the risk of radiation-induced liver disease, with limited evidence and no consensus on optimal dose, fractionation, and scheduling. Lee P et al. found that SBRT is a viable option for carefully selected patients with Child-Pugh scores B7-C10, based on a small cohort study [14]. The study reported no significant differences in local control or overall survival based on baseline Child-Pugh score. Twenty-three patients with Child-Pugh B/C were treated with SBRT, with a median tumor size of 3.1 cm and a median dose of 40 Gy delivered in 5 fractions. Culleton S et al. reported on 29 patients with Child-Pugh B/C HCC treated with SBRT, receiving a median dose of 30 Gy in 6 fractions. The clinical outcomes from this study indicated a median survival of 7.9 months [15].

SBRT allows for high-dose radiation to be delivered to the tumor while sparing surrounding healthy liver tissue, making it particularly useful for patients with compromised liver function. In this case, the large tumor size exceeding 5 cm further complicates treatment. Conventionally, larger tumors are associated with poorer outcomes, and there is an increased risk of tumor progression during the waiting period for transplantation. By employing SBRT, the aim is to achieve local tumor control, prevent progression beyond transplant criteria, and improve post-transplant outcomes. Further research is needed to better define the long-term outcomes of SBRT as a bridging therapy for HCC patients with severe cirrhosis, including its impact on post-transplant survival and liver function recovery. Prospective studies comparing SBRT with other bridging therapies in this high-risk population would also be beneficial.

## Conclusions

In this case, SBRT provided an effective bridge to transplantation for a patient with HCC larger than 5 cm and Child-Pugh Class C cirrhosis. It offers a valuable treatment option for achieving local tumor control and preventing tumor progression in patients with limited liver function, although careful patient selection and close monitoring for potential liver toxicity are essential. SBRT represents a promising therapy for this challenging patient population and can play a critical role in expanding the eligibility of patients with advanced HCC for liver transplantation.
